# Cost-Effectiveness Analysis of a Machine Learning–Based eHealth System to Predict and Reduce Emergency Department Visits and Unscheduled Hospitalizations of Older People Living at Home: Retrospective Study

**DOI:** 10.2196/63700

**Published:** 2025-04-11

**Authors:** Charlotte Havreng-Théry, Arnaud Fouchard, Fabrice Denis, Jacques-Henri Veyron, Joël Belmin

**Affiliations:** 1 Laboratoire Informatique Médicale et Ingénierie des Connaissances en eSanté, Institut National de la Santé et de la Recherche Médicale and Sorbonne Université Paris France; 2 PRESAGE Paris France; 3 EIR Conseil Santé Antony France; 4 Institut Inter-Régional de Cancérologie Jean Bernard Le Mans France; 5 Hôpital Charles Foix, Assistance Publique-Hôpitaux de Paris Ivry-sur-Seine France

**Keywords:** monitoring, older adult, predictive tool, home care aide, emergency department visit, cost-effectiveness, artificial intelligence, electronic health, eHealth, emergency department, unscheduled hospitalization, aging, retrospective study, medico-economic, living at home, nursing home, emergency visit, Brittany, France, machine learning, remote monitoring, digital health, health informatics

## Abstract

**Background:**

Dependent older people or those losing their autonomy are at risk of emergency hospitalization. Digital systems that monitor health remotely could be useful in reducing these visits by detecting worsening health conditions earlier. However, few studies have assessed the medico-economic impact of these systems, particularly for older people.

**Objective:**

The objective of this study was to compare the clinical and economic impacts of an eHealth device in real life compared with the usual monitoring of older people living at home.

**Methods:**

This study was a comparative, retrospective, and controlled trial on data collected between May 31, 2021, and May 31, 2022, in one health care and home nursing center located in Brittany, France. Participants had to be aged >75 years, living at home, and receiving assistance from the home care service for at least 1 month. We implemented among the intervention group an eHealth system that produces an alert for a high risk of emergency department visits or hospitalizations. After each home visit, the home care aides completed a questionnaire on participants’ functional status using a smartphone app, and the information was processed in real time by a previously developed machine learning algorithm that identifies patients at risk of an emergency visit within 7 to 14 days. In the case of predicted risk, the eHealth system alerted a coordinating nurse who could then inform the family carer and the patient’s nurses or general practitioner.

**Results:**

A total of 120 patients were included in the study, with 60 in the control group and 60 in the intervention group. Among the 726 visits from the intervention group that were not followed by an alert, only 4 (0.6%) resulted in hospitalizations (*P*<.001), confirming the relevance of the system’s alerts. Over the course of the study, 37 hospitalizations were recorded for 25 (20.8%) of the 120 patients. Additionally, of the 120 patients, 9 (7.5%) were admitted to a nursing home, and 7 (5.8%) died. Patients in the intervention group (56/60, 93%) remained at home significantly more often than those in the control group (48/60, 80%; *P*=.03). The total cost of primary care and hospitalization during the study was €167,000 (€1=US $1.09), with €108,000 (64.81%) attributed to the intervention group (*P*=.20).

**Conclusions:**

This study presents encouraging results on the impact of a remote medical monitoring system for older adults, demonstrating a reduction in both emergency department visits and hospitalization costs.

**Trial Registration:**

ClinicalTrials.gov NCT05221697; https://clinicaltrials.gov/study/NCT05221697

## Introduction

### Older Adults and Emergency Hospitalizations

In France, over 13.4 million people are aged 65 years or older, representing 20% of the French population. This proportion has increased by 4% in 20 years [1]. With aging and frailty, the risk of unplanned hospitalizations is increased.

Studies show that in France, older adults living at home represent 13% of the population that is hospitalized at least once a year, compared to 6% for the general population [2,3]. According to medico-economic data, nearly 1.6 million people aged >80 years were hospitalized in 2017, twice the number in the general population. In addition, the average length of stay doubles between the ages of 60 and 80 years, reaching nearly 10 days for those aged >90 years [4].

Depending on the study, between 19% and 67% of hospitalizations of older people are considered avoidable [5,6].

Medical and social frailty and comorbidity of the older population are the main factors that increase the prevalence and time spent in hospital [3].

The emergency department (ED) is the main entry point for the older people. Half of the people who visit the ED are subsequently hospitalized. Moreover, older adults represent 41% of ED visits, and nearly 23% of them spend more than 8 hours there and 34% spend between 4 and 8 hours—more than twice as long as younger people. This longer stay in the ED is compounded by a higher rate of transfer to other departments or hospitals [3,7].

In people aged >80 years, main causes of admission to the ED are traumatic events (generally related to fall; 25%), cardiovascular events (17%), altered general condition or infection (12%), a respiratory symptom (12%), a gastrointestinal symptom (10%), and neurological symptoms (9%) [3,7].

Emergency hospitalization represents a significant medical and economic cost. Older adults’ hospitalizations reduce the level of dependency by 30% to 60% (immobilization syndrome, confusion, malnutrition, urinary incontinence, undesirable effects related to treatments, etc). While people aged >75 years represent 8% of the population, they are responsible for one-fifth of health care expenditure [8,9]. In France, the main expenses to support the loss of autonomy of older adults are approximately €12.2 billion (€1=US $1.09) in care expenses and €10.7 billion in human and technical assistance. The challenge is not so much to reduce hospitalization rates or the average length of stay but rather to avoid preventable emergency hospitalizations [1,2,8,10,11].

### Telehealth and Artificial Intelligence for the Prediction and Prevention of Emergency Situations in Older Adults

Therefore, it is crucial to anticipate adverse events at home. The use of eHealth systems, telemedicine, and connected objects represent a promising approach for keeping older adults at home and preventing the loss of autonomy. Patient-reported outcome measures benefit people with chronic diseases by improving their quality of life, reducing mortality, and reducing ED visits and hospitalizations [7,12].

We have implemented a machine learning–based eHealth device to predict and prevent ED visits and unscheduled hospitalizations for older people living at home [13,14]. In 2022, we conducted a multicenter study with people aged >65 years, living at home, and receiving regular visits of home care aides (HCAs) to evaluate this device in real life. After each home visit, HCAs completed a smartphone-based questionnaire on the functional status of the patients. The information was processed in real time by a machine learning algorithm developed beforehand in order to identify patients at risk of an ED visit within 7 to 14 days (with a predictive performance of 83% sensitivity and 86% specificity). This machine learning algorithm can also predict symptoms or events such as malnutrition, falls, swollen legs, or depressions [15]. In the case of predicted risk, the system alerted a nurse coordinator who could then inform the family caregiver and the patient’s nurses or general practitioner. A total of 206 patients were included and followed for 10 months. Compliance was good. Among the 2656 home visits, 405 alerts were issued. The system significantly reduced the number of ED visits when an intervention was performed after an alert. The system was considered easy to use and useful, and it was well accepted by more than 90% of the HCAs and coordinating nurses [15].

This algorithm opens the possibility of mobilizing health professionals to intervene early in an acute illness or in the decompensation of a chronic illness before it leads to an emergency hospitalization.

### Objectives

The main hypothesis of this study was as follows: using a simple tool to predict the risk can allow HCAs to anticipate the incidence of emergency hospitalizations and, thereby, reduce the cost and side effects of emergency hospitalization.

The objective of this study was to analyze the clinical and economic impacts of this eHealth device in real life compared to the usual monitoring of frail older people living at home. In France, as this medical device is the first to predict ED use, there are, to our knowledge, few medico-economic studies available.

## Methods

### Study Design

This study was a single-center, retrospective, and controlled trial on data collected between May 31, 2021, and May 31, 2022, in one health care and home nursing center located in Brittany, France.

### Recruitment

Participants were recruited from among adults aged 75 years and older who were living at home and receiving the assistance from HCAs. These HCAs were not health care professionals and typically provided assistance with nonmedical tasks (eg, helping with meals, assisting with personal care, housekeeping, and running errands). The dependency levels of the persons were established according to the French national instrument, which stratifies the dependency level from *groupe iso-ressources* (GIR) 1 (very severe dependency) to GIR 6 (no dependency) [16].

Patients in palliative care situations (GIR 1) were not included. Patients were proposed to be included in the controlled group without being offered any incentives. Written consent was obtained from all participants of the intervention group to be included in the study.

The control group was composed of people receiving the usual support from the home care service. The adjustment between intervention and control groups (1:1 or 1:2 when possible) was based on age (within 2 years), gender, and level of dependency. Participants in the control group were informed of their enrollment in the study. Data were collected between May 30, 2021, and May 31, 2022.

### Data Collection

Sociodemographic and pathway data—family situation, dependency level, ED visits (dates and causes), hospitalization (dates and causes), and death (date)—were collected via the tracking system (for the intervention arm) and the coordinating nurses (for the control arm).

### Intervention

For participants in the intervention group, HCAs used a smartphone app, for which they received training on its use (a 1-hour session at the start of the study). Each week, they completed a simple and concise questionnaire for each patient via the app. This questionnaire comprised 25 yes-or-no questions covering functional and clinical autonomy (activities of daily living); medical symptoms such as fatigue, falls, pain, and undernutrition; as well as behavioral changes (cognitive disorders and aggression), communication with caregivers, and social life.

The collected data were transmitted in real time to a secure server, where an artificial intelligence algorithm analyzed them to assess risk levels and predict ED visits within 7 or 14 days. The risk assessment was displayed on a Conformité Européenne–marked, web-based secure medical platform called PRESAGE CARE. When the algorithm detected a high-risk level, an on-screen alert was sent to the nurse coordinator of the home care center. This alert included information on recent changes in the patient’s functional status, along with decision-support insights to help the nurse coordinator take appropriate action, such as contacting the family caregiver or other health care professionals, or conducting a home reassessment.

No specific intervention protocol was imposed on health care professionals, allowing them full autonomy in their decision-making. This alert-based intervention model was presented to and approved by the *Agence du Numérique en Santé* (National Agency for eHealth). The study followed the guidelines and recommendations of the *Haute Autorité de Santé* (French Health Authority) regarding the cost-effectiveness evaluation of medical devices.

### Outcomes

The primary outcome was the difference in the cumulative incidence of emergency hospitalizations between the two groups, with death and institutionalization considered competing events. Unscheduled hospitalizations were defined as those occurring after an ED visit or those explicitly recorded as such in the PRESAGE CARE console or the data extracted from the center’s information system.

Secondary outcomes included a comparison of hospitalization rates following an alert that did not lead to an intervention versus those following an alert that triggered an intervention, as well as a cost comparison of hospitalizations between the two groups. Additionally, the probability of remaining at home was assessed, defined as the time elapsed from May 30, 2021 (study start date), to the first occurrence of one of the following events: unplanned hospitalization, death, or institutionalization.

### Cost Analysis

For each group, the hospital costs attributable to the ED visits and hospitalizations (unplanned and planned) were calculated on the basis of the homogeneous group of patients, determined by the declared causes of the hospitalization (CIM-10 PMSI coding, which is the French adaptation of US diagnosis-related groups). Hospital costs have been determined on the basis of published costs by the type of illness and length of hospital stay (basic category of classification in medicine, obstetrics, or surgery). The costs of consultations with the attending physician related to the PRESAGE CARE system were also included. The average length of hospital stay was compared between the groups. The burden costs (gross salary and taxes) of nurses who managed alerts (intervention arm) and conducted regular activities (control arm) were defined according to the time needed to proceed their tasks and average gross salary for nurses [17].

The cost for data collection by HCAs was defined as training costs (1 hour of training time multiplied by the hourly burden costs for HCAs [17]). The collection of data into PRESAGE Care eHealth device occurred during regular visits so no extra time was counted. In this study, the medical device was deployed free of charge.

The analysis was performed from the point of view of the total hospital cost per event and per patient in relation to the medical benefit determined by the number of deaths, the number of institutionalizations, and the data in the literature concerning the loss of functional independence following hospitalization (in the absence of data on the reassessment of the dependency level after hospitalization). The cost analysis was mainly carried out from the point of view of social security expenditure.

Coordinating nurses were asked to report time spend in order to check alerts and act according to the procedure. The number of ED visits, emergency hospitalizations, deaths, and nursing home admissions were compared between the groups. The times before the first event were compared between groups.

### Statistical Analysis

Continuous variables were described in terms of means, medians, IQRs, and SDs, depending on the normality of the distribution. Categorical variables were described in terms of numbers and percentages. Groups were compared using the chi-square or Fisher test for categorical variables and the Wilcoxon or 2-tailed *t* test for quantitative variables. Comparisons of cumulative incidences were made by the Gray test (entry into an institution and death in competition). A value of *P*<.05 was considered significant. Statistical analyses were performed using Stata software (version 17; StataCorp LLC).

### Ethical Considerations

The research protocol for this study was submitted to and approved by the French National Committee for Biomedical Research, the Committee for the Protection of Individuals, and the French Agency for Health Product Safety (registration: 2234275). The beneficiaries as well as the caregivers and professionals involved were informed of the nature of this study and gave their written consent. Privacy and data protection rules were presented to participants and are available upon request. No compensation was offered.

## Results

### Patients’ Characteristics

A total of 120 patients were included in the study, with 60 (50%) in the control group and 60 (50%) in the intervention group. Among them, 86 (71.7%) were women, including 45 (75%) of the 60 patients in the intervention group and 41 (68%) of the 60 patients in the control group (*P*=.76). The mean age of participants was 83.77 years, with no significant difference between the groups (*P*=.44).

Dependency levels, assessed using the *autonomie gérontologique GIR* grid, showed an average dependency score of 3.79, with no significant differences between the groups (all *P*>.05; Table 1). Overall, the characteristics of participants in the intervention and control groups were comparable.

**Table 1:** Participants’ characteristics.

Characteristics	Intervention group (n=60)	Control group (n=60)	*P* value
Age (years), mean (95% CI)	84.55 (81.78-87.32)	82.99 (80.08- 85.90)	.44
Female, n (%)	45 (75)	41 (68)	.42
Low dependency level (GIR^a^ 5-6), n (%)	14 (23)	10 (17)	.36
Mild dependency level (GIR 3-4), n (%)	37 (62)	37 (62)	>.99
High dependency level (GIR 2), n (%)	9 (15)	8 (13)	.79

^a^GIR: *groupes iso-ressources*.

In the intervention arm, a total of 792 visits were monitored through the app. Among these visits, 66 alerts were issued and transmitted to a coordinating nurse, of which 21 (32%) led to a health intervention (such as a general practitioner visit or a home visit by a nurse).

Following an alert-triggered intervention, only 1 (5%) out of the 21 patients was hospitalized, compared to 10 (91%) out of the 11 patients when no intervention was performed after an alert (*P*<.001). Among the 726 visits that did not trigger an alert, 4 (0.6%) hospitalizations occurred (*P*<.001; Table 2).

**Table 2:** Alert-triggered interventions and hospitalizations.

			Total		No hospitalization		Hospitalizations		*P* value	
**Alerts (n=66)**										<.001
	No alert-triggered intervention, n (%)	45 (68)		35 (78)		10 (22)				
	Alert-triggered intervention, n (%)	21 (33)		20 (95)		1 (5)				
**No alerts (n=726), n (%)**			726 (100)		722 (99.4)		4 (0.6)		<.001	

### Medical impact

Altered general condition (7/37, 19%) and falls with fractures (7/37, 19%) were the most frequently described cause, followed by dyspnea (6/37, 16%). The causes are relatively distributed (Table 3).

**Table 3:** Unplanned hospitalizations causes (n=37).

Causes	Total, n (%)	Intervention group, n (%)	Control group, n (%)
Altered general condition	7 (19)	1 (3)	6 (16)
Dyspnea	6 (16)	2 (5)	4 (11)
Fall with fracture	7 (19)	4 (10)	3 (8)
Emergency surgery	5 (14)	2 (5)	3 (8)
Acute pain	4 (11)	2 (5)	2 (5)
Psychological symptoms	2 (5)	0 (0)	2 (5)
Kidney failure	2 (5)	1 (3)	1 (3)
Heart failure	1 (3)	0 (0)	1 (3)
Stroke	1 (3)	1 (3)	0 (0)
Fall without fracture	1 (3)	1 (3)	0 (0)
Missing value	1 (3)	1 (3)	0 (0)
Total	37 (100)	15 (41)	22 (59)

#### Emergency Hospitalizations, Deaths, and Nursing Home Admissions

A total of 37 emergency hospitalizations were recorded during the study, involving 25 patients. The number of emergency hospitalizations was 32% lower in the intervention group (15/37, 41%) compared to the control group (22/37, 59%).

Of the 6 scheduled hospitalizations, 5 occurred in the intervention group. The cumulative incidence of emergency hospitalizations was 23.3% (95% CI 13.5%-34.7%) in the control group, compared to 16.7% (95% CI 8.5%-27.2%) in the intervention group (*P*=.37).

Among participants, 16 patients left their homes due to either admission to a nursing home (9/120, 7.5%) or death (7/120, 5.8%). There were 7 deaths: 1 (14%) in the intervention group and 6 (86%) in the control group. Nine patients were admitted to a nursing home, including 3 (33%) in the intervention group and 6 (67%) in the control group.

The proportion of patients who left home was significantly higher in the control group (12/16, 75%) than in the intervention group (4/16, 25%; *P*=.03; Table 4).

**Table 4:** Health events during the study.

Health events			Total (N=120)		Intervention group, (n=60)		Control group, (n=60)		Difference between groups (%)		*P* value
**In terms of the number of events (n=37), n (%)**											
	Emergency hospitalizations	37 (100)		15 (40.5)		22 (59.5)		–32		.16	
**In terms of the number of patients, n (%)**											
	Emergency hospitalizations	25 (20.8)		10 (16.7)		15 (25)		–33		.26	
	Deaths	7 (5.8)		1 (1.7)		6 (10)		–67		.05	
	Entry into nursing homes or facilities	9 (7.5)		3 (5)		6 (10)		–50		.30	
	Patients who left home	16 (86.7)		4 (6.7)		12 (20)		–67		.03	

#### Probability of Staying at Home

The survival analysis for the probability to stay at home was 68% (95% CI 54.5%-78.3%) for the control group versus 80% (95% CI 67.5%-88.1%) for the intervention group (*P*=.15).

### Costs

#### Cost of Follow-Up

Eleven individuals benefited from a consultation with a general practitioner following an alert issued by the PRESAGE CARE system. These consultations were considered additional costs attributable to the use of the device. The time required for nurses to monitor patients in the intervention arm totaled 119 hours (equivalent to 2 minutes and 40 seconds per patient per week), resulting in a total burden cost of €3460 [16]. Training for HCAs was conducted in a group setting over 1 hour, representing a cost of €1334 for 30 HCAs. Training for the coordinating nurse lasted 5 hours, with an associated cost of €200. The total cost related to training time and device usage was estimated at €4994.

#### Hospital Costs

**Figure 1:**
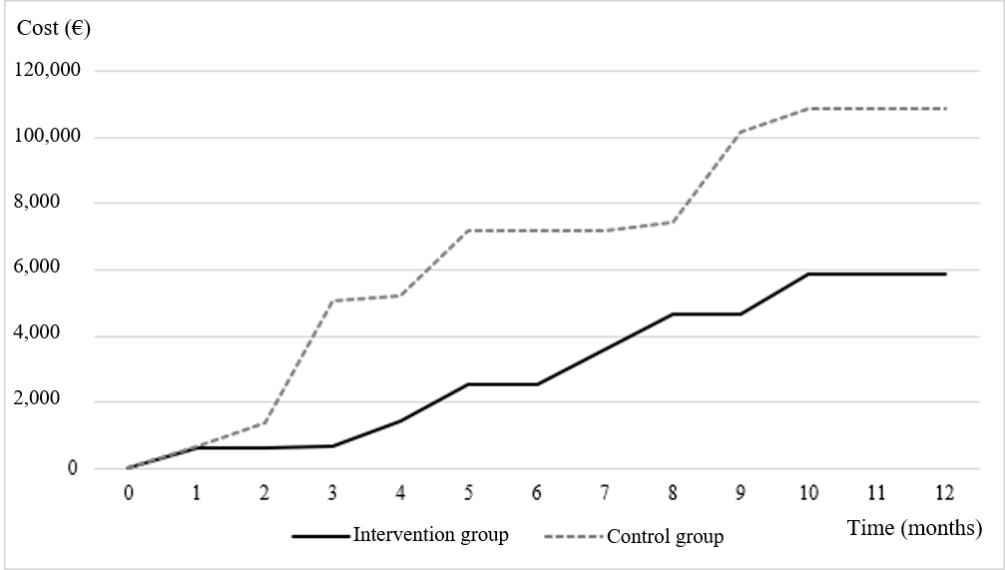
Emergency hospitalizations' costs evolution (cumutive cost, 1€ = US $ 1.09).

All causes of hospitalization and their durations were recorded and categorized according to the relevant homogeneous group of patients. The median length of stay per hospitalization was 9.8 (IQR 4.73-14.87) days in the intervention group, compared to 13.61 (IQR 6.70-20.52) days in the control group, representing a 67% difference. Over the course of the year, the total number of hospitalization days was 6 times higher in the control group than in the intervention group, with 882 days versus 147 days, respectively. The annual cost attributed to emergency hospitalizations, based on data from the homogeneous group of patients, was €58,975 in the intervention group compared to €108,642 in the control group, reflecting a 45.72% reduction in the intervention group (Figure 1). The total cost for the intervention group was €64,300, compared to €108,642 in the control group, representing a 40.82% cost reduction (Table 5).

**Table 5:** Costs analyses.

	Intervention group	Control group	Difference between groups (%)	*P* value
Length of stay (days), median (IQR)	9.8 (4.73-14.87)	13.61 (6.70-20.52)	–28	.41
Total number of days of emergency hospitalizations, n	147	313	–53	.09
Annual emergency hospitalization costs (€)^a^	58,976	108,641	–45	.20
Cost for additional consultations with the attending physician (€)	330	—^b^	—	
Cost for data collection (training cost for HCAs^c^; €)	1334	—	—	
Cost for coordinating nurse (follow-up and alert management; €)	3460	—	—	
Cost for coordinating nurse (training cost; €)	200	—	—	
Total annual cost (€)	64,300	108,641	–41	.25
Mean cost by patient (€)	1071	1810	—	

^a^€1=US $1.09.

^b^Not applicable.

^c^HCA: home care aide.

## Discussion

### Principal Findings

The objective of this study was to assess the medico-economic impact of an eHealth device designed to predict and prevent emergency hospitalizations among frail older adults living at home.

The use of the PRESAGE CARE device led to a nearly 32% reduction in emergency hospitalizations compared to the control group. Overall, it reduced hospital-related costs by 45.72%. In addition, this cost reduction must be considered in the context of additional expenses, including in-home care, posthospitalization consultations, and the increased risk of rehospitalization.

The median length of stay in the intervention group was consistent with findings in the literature, whereas it was higher in the control group, likely due to extreme values and the small cohort size.

Furthermore, over a 1-year follow-up of 60 participants, the system extended the mean time to failure in the care pathway by 38 days. This result is likely underestimated given the limited cohort size.

In the intervention arm, among the 726 visits not followed by an alert, only 4 (0.6%) hospitalizations followed the visit (*P*<.001), which confirm the relevance of the alerts issued by the system. Comparison of the postintervention hospitalization rate in the intervention group shows that an alert-triggered intervention significantly reduces hospitalization. These results are consistent with a previous study on this system [15]. Professionals can effectively anticipate a deteriorating health condition and act at the right time.

The multiplication of technological tools to promote home care rarely benefit from data on their real medico-economic benefits [17,18]. Moreover, few studies on the economic evaluation of telemedicine systems show a medico-economic gain [19-22]. In addition, to our knowledge, they have not evaluated the effectiveness of a predictive system for the prevention of unplanned hospitalizations of older people living at home.

### Cost of the Device

The cost of the device has not been taken into account in this study as it is currently being evaluated in terms of its impact. The price of the health care sector (including hospital care) varies widely from one country to another. France remains 40% cheaper than the United States and 55% cheaper than Switzerland [23].

### Increased Cost of Dependency and Quality of Life

According to the report of the Hospital and Older People Workshop published in 2018, the incidence of dependence related to hospitalization varies between 30% and 60% in people aged 70 years and older and increases to 50% in patients aged 85 years and older. After discharge from hospital, only 50% of patients recover their basic functional state, 33% within 6 months after discharge, and 14% at 1 year [10,19]. The prevalence of iatrogenic dependence is about 12%, and it is avoidable in 80% of cases. It is therefore possible to prevent it.

Moreover, after a visit to the ED, 56% of older patients are hospitalized, and 44% return home. The difficulty in finding suitable hospital places for older adults leads to an increase in the time spent by carers on calls to other departments. Older people spend more time in the ED, on average 4 hours more [10].

These data increase the medical and financial impact of devices aimed at predicting and preventing ED visits and emergency hospitalizations.

The increase in the loss of autonomy of older adults after hospitalization (bed rest, loss of mobility, and waiting time in the ED) leads to an increase in home care needs and increases the costs associated with the management of dependency.

This increase in posthospitalization dependence leads to a decrease in quality of life, which has been widely described in the literature [24].

This device represents a real gain in terms of medical benefits, quality of life, and hospital costs.

### Limitations

Our study has several limitations. First, because the overall study design was not randomized at the outset of the study, there is a bias in the allocation of groups. Moreover, the number of deaths was higher in the control group, but the sample size did not allow us to identify a significant correlation with the intervention. The lack of significance of the different results can indeed be explained by the size of the cohort, the number of events, and the low statistical power. Some particularly long hospital stays imply a wide CI, impacting the significance of the results.

In view of the number of deaths and the lack of consensus on the relevance of using quality-adjusted life-year analyses to carry out medico-economic studies of dependent or frail older people (National Academy of Medicine) [21,24], it was not possible to carry out a quality-adjusted life-year study based on life expectancy.

Furthermore, the quality of life of the individuals could not be measured and compared. It is not possible to draw conclusions on the impact of PRESAGE CARE on the quality of life of beneficiaries. A prospective study will be conducted to evaluate this impact.

The size of the cohort is also a limitation and will require a larger-scale, multicenter, randomized controlled trial.

### Conclusion

This first study evaluating the medico-economic impact in real life of the use of the PRESAGE CARE medical device shows a reduction of almost half of the hospital expenses among the beneficiaries of the device. In addition, PRESAGE CARE reduced unplanned hospitalizations by more than 30% and increased life expectancy in good health at home. A larger, randomized controlled trial is needed to confirm these already very encouraging results.

## Abbreviations

ED: emergency department
GIR: groupe iso-ressources
HCA: home care aide

## Data Availability

The data that support the findings of this study are available from the corresponding author, JHV, upon reasonable request. Restrictions apply to the availability of the data collected by the home care aids and the algorithm that was used in this study, which is the subject of a patent.
